# Identification and *In Silico* Analysis of a Homozygous Nonsense Variant in *TGM1* Gene Segregating with Congenital Ichthyosis in a Consanguineous Family

**DOI:** 10.3390/medicina59010103

**Published:** 2023-01-02

**Authors:** Abdulhadi Almazroea, Ambreen Ijaz, Abdul Aziz, Muhammad Mushtaq Yasinzai, Rafiullah Rafiullah, Fazal Ur Rehman, Shakeela Daud, Rozeena Shaikh, Muhammad Ayub, Abdul Wali

**Affiliations:** 1 Pediatrician, Associate Professor at College of Medicine, Taibah University, Madinah 41477, Saudi Arabia; 2 Department of Biotechnology, Faculty of Life Sciences & Informatics, BUITEMS, Quetta 87300, Pakistan; 3 Department of Zoology, SBK Women’s University, Quetta 87500, Pakistan; 4 Department of Computer Sciences and Bioinformatics, Khushal Khan Khattak University, Karak 27200, Pakistan; 5 Department of Microbiology, University of Balochistan, Quetta 87550, Pakistan; 6 Institute of Biochemistry, University of Balochistan, Quetta 87550, Pakistan

**Keywords:** lamellar ichthyosis, *TGM1*, nonsense mutation, *in silico* analysis

## Abstract

*Background and Objectives:* Lamellar ichthyosis is a rare skin disease characterized by large, dark brown plate-like scales on the entire body surface with minimum or no erythema. This phenotype is frequently associated with a mutation in the *TGM1* gene, encoding the enzyme transglutaminase 1 which plays a catalytic role in the formation of the cornified cell envelop. The present study aimed to carry out clinical and genetic characterization of the autosomal recessive lamellar ichthyosis family from Balochistan. *Materials and Methods:* A consanguineous family with lamellar ichthyosis was enrolled from Balochistan, Pakistan. PCR amplification of all the exons and splice site junctions of the *TGM1* gene followed by Sanger sequencing was performed on the genomic DNA. The identified variant was checked by *In silico* prediction tools to evaluate the effect of the variant on protein. *Results:* Sanger sequencing identified a homozygous nonsense variant c.131G >A (p.Trp44*) in the *TGM1* gene that segregated in the autosomal recessive mode of inheritance in the family. The identified variant results in premature termination of transcribed mRNA and is predicted to cause a truncated or absent translation product transglutaminase-1 (TGase-1) accompanied by loss of catalytic activity, causing a severe clinical phenotype of lamellar ichthyosis in the patients. *Conclusions:* Here, we report a consanguineous lamellar ichthyosis family with a homozygous nonsense variant in the *TGM1* gene. The variant is predicted as pathogenic by different *In silico* prediction tools.

## 1. Introduction

Congenital ichthyosis is a rare genetic disorder which is phenotypically and genetically heterogeneous and inherited in an autosomal recessive manner. It is characterized by generalized involvement of skin in the form of hyperkeratosis, widespread scaling and dryness caused by abnormal differentiation of epidermal cells [1,2]. The latest classification of ichthyosis differentiates two main categories as syndromic and nonsyndromic forms.

Syndromic ichthyosis is presented with clinical manifestations of skin along with other organs whereas nonsyndromic ichthyosis patients have only generalized involvement of skin [3]. Ichthyosis syndromes can be either X linked ichthyosis (XLI) or autosomal. XLI syndromes have multiple clinical symptoms comprising hyperkeartosis, short stature, mental retardation, and ocular albinism. Recessive X-linked ichthyosis (RXLI) syndrome such as ichthyosis follicularis, cornadi hunermann-happle syndrome, and atrichia photophobia syndrome (IFAP) are familiar subtypes of XLI [4,5]. Autosomal ichthyosis syndromes are further categorized on the basis of lethal clinical signs, pronounced neurological complications, skeletal and muscular abnormalities, as well as prominent hair deformities. Five of the subtypes with prominent hair abnormalities are Netherton syndrome, Trichothiodystrophy, Ichthyosis Hypotrichosis Syndrome, Ichthyosis Hypotrichosis-Sclerosing Cholangitis, and Ichthyosis Follicularis–Atrichia–Photophobia Syndrome [6]. Refsum syndrome, Sjogren-Larsson syndrome (SLS), Gaucher syndrome type 2, and multiple sulphatase deficiency (MSD) Refsum syndrome are also reported as lethal types of autosomal syndromes [4].

Nonsyndromic ichthyosis includes recessive X-linked ichthyosis (RXLI), ichthyosis vulgaris (IV), congenital ichthyosiform erythroderma (CIE), harlequin ichthyosis (HI), lamellar ichthyosis (LI), and keratinopathic ichthyosis (KI), self-improving collodion ichthyosis (SICI) and bathing suit ichthyosis (BSI). IV and RXLI are considered as common forms whereas LI, HI, and CIE are the rarest types and collectively called autosomal recessive congenital ichthyosis (ARCI) [4,7]. IV is the common sub type of ichthyosis with the prevalence of 1:100 [8]. It is characterized by thin pale grey scales on the body surface except articular flexures and palmo-plantar hyperlinearity [9]. It is caused due to loss of function mutation in the gene filaggrin (*FLG*), inherited in an autosomal dominant pattern. *FLG* comprises three exons and is mapped on chromosome 1q21 [10], encoding the enzyme filaggrin. Filaggrin plays a crucial part in the formation of the skin barrier and helps in terminal differentiation of epidermal cells [11]. More than 90 pathogenic mutations have been reported in the *FLG* in patients with clinical signs of IV and atopic dermatitis [12]. XRI has the prevalence of 1 in 2000 boys [7] who suffer scaling at the upper trunk, extensor parts of the extremities, and posterior part of the neck since infancy [13,14]. It is caused due to a loss of the enzyme steroid sulphatase (STS), encoded by the *STS* gene which is mapped at the short arm of chromosome X. [15]. Most of RXLI cases (90%) have reported large deletion mutations [9]. This gene is highly expressed in the skin, liver, lymph nodes, and placenta, as well as in brain and breast tissues [16].

ARCI is a rare heterogeneous group of disorders of keratinization, characterized mainly by hyperkeratosis, variable erythema, and abnormal skin desquamation. Defects in the intracellular lipids of epithelium are responsible for clinical manifestations of ARCI [17]. ARCI have three clinical sub classes: Congenital ichthyosiform erythroderma (CIE) (MIM 242100), Lamellar ichthyosis (LI) (MIM 242300), and Harlequin ichthyosis (HI) (242500) [18]. Both CIE and LI affected babies are born with a stiff, transparent, and glossy thick collodion membrane, which is unwrapped and dries during the few days after birth. CIE babies, after the removal of the collodion membrane, show variable erythroderma, having finer white and reddish scales all over the surface of the body. LI affected individuals show thickened, large, plate-like dark, dry scaling with absent or lesser erythema on the body surface associated with scarring alopecia, prominent palmoplantar keratoderma, eclabium, and ectropion [19,20]. HI is the most fatal and rare subtype subjected to neonatal death. These patients are born prematurely, covered with hyperkeratotic plaques causing movement constraint resulting in lethal respiratory problems [20,21]. Some of the reported ARCI patients showed the clinical symptoms of both CIE and LI [22]. Mutation screening studies have found that LI and CIE have been caused by different mutations in one gene [23].

Up to date, fourteen genes have been reported to be associated with ARCI phenotypes; these are arachidonate 12-lipoxygenase R type (*ALOX12B*, MIM: 603741), transglutaminase 1 (*TGM1*, MIM:190195), arachidonate lipoxygenase 3 (*ALOXE3*, MIM:6-7206), Nipa-like domain-containing 4 (*NIPAL4*, MIM: 609383), Patatin-like Phospholipase domain-containing protein 1 (*PNPLA1*, MIM: 612121), Short Chain Dehydrogenase Reductase Family 9C member 7 (*SDR9C7*, MIM: 609769), ATP-binding cassette subfamily A member 12 (*ABCA12*, MIM: 607800), cytochrome p450 family 4 subfamily F polypeptide 22 (*CYP4F22*, MIM: 611495), Solute carrier family 27 fatty acid transporter member 4 (*SLC27A4*, MIM: 604194), Caspase 14 Apoptosis-related cysteine protease (*CASP14*, MIM: 605848), lipase family member N (*LIPN*, MIM:613924), ST14 transmembrane serine protease matriptase, sulfotransferase family 2B member 1 (*SULT2B1*, MIM: 604125), and ceramide synthase 3 (*CERS3*, MIM: 615276) [24,25,26,27]. Among these, LI phenotype is reported to be caused by mutations in *ALOXE3*, *ABCA12*, *ALOX12B*, *NIPAL4*, *CYP4F22*, *CERS3*, *PNPLA1*, and *TGM1* genes [28]. *ALOXE3* and *ALOX12B* mutations have been identified in 17% of ARCI cases [29]. Both the *ALOXE3* and *ALOX12B* are located on chromosome 17q13.1 and possess similar structures, comprising 15 exons [30]. *ALOXE3* and *ALOX12B* are localized on chromosome 17q13.1, encoding enzymes eLOX-3 and 12R-LOX, respectively, which function in the catalysis of arachidonic acid pathways for epidermal differentiation, development of lamellar bodies, and their transfer to the extracellular space [11,31,32]. *ABCA12* is a large gene consisting of 53 exons and mapped to chromosome 2q34 [33]. It functions as transmembrane lipid transporter required for multistep metabolism of lipid contents of lamellar granules and making an intercellular lipid layer around keratinocytes for epidermal barrier function [17,34]. Genetic defects in *ABCA12* are reported to cause LI and HI phenotypes depending on the nature of mutation. Homozygous missense *ABCA12* mutations have been reported in severe LI cases whereas loss of function mutations were identified as genetic causes of HI [4,35,36]. *NIPAL4* is located on chromosome 5q33 and comprises six coding exons [37]. Its translation product is known as ichthyin which functions as a receptor molecule for certain ligand substances such as troxillin A3 and B3 in hepoxilin metabolic pathways to help in the formation of lamellar bodies and their transport [37]. *NIPAL4* has been reported in 16% of ARCI patients in literature [38]. The *CYP4F22* gene has 12 exons and is located at chromosome 19p13.2; it encodes the protein belonging to the super family of CYP heme-thiolate enzymes. It functions in the biochemical pathway of arachidonic acid metabolism and synthesis of eicosanoid required for skin barrier formation [39,40]. ARCI symptoms due to causative mutations in *CYP4F22* have been identified in 8% of cases [37,41]. CERS3 has 15 exons, located at chromosome 15q26.3 and belongs to the ceramide synthase family of genes. The encoded ceramide synthase enzyme catalyzes the formation of ceramides from a sphingoid base and acyl-coA substrates and controls sphingolipid synthesis required in the epidermis to perform as a protective barrier [42,43]. Mutations in the *CERS3* gene causes defective epidermal differentiation leading to the disruption of skin barrier function [9]. The gene *PNPLA1* is located on chromosome 6p21, having eight exons [44]. It encodes the enzyme PNPLA1 which functions as transacylase. It is responsible for the formation of acylceramides, essential for skin barrier function. Mutations in *PNPLA1* cause deficiency of omega-O-acylceramides to effect the lipid barrier of the epidermis [45,46,47]. Causative nonsense and missense in *PNPLA1* have been identified in CIE families from Algeria and Morocco [44]. Other PNPLA1 cases have been reported in families from Spain, Pakistan, Iran, and northern Europe [48,49,50,51].

The *TGM1* Gene comprises of 15 exons, and encodes an enzyme TGase-1, located on chromosome 14q11.2. The molecular weight of the TGase-1 enzyme is 89 kDa and consists of 817 amino acids [52,53,54]. It is reported to be associated with the majority of LI cases [27,55,56]. It encodes the enzyme TGase-1, expressed in the epidermis of the skin; presents in the plasma membrane and catalyzes cross-linking of proteins; and is necessary for the production of the cornified cell envelop (CCE) of the stratum corneum [57,58]. The cornified barrier protects the skin from dehydration and detrimental effects of pathogens by forming the envelop [59]. The leading causes of the LI phenotype are nonsense and missense mutations in *TGM1* that inactivates the TGase-1 enzyme and formation of CCE [60,61]. Despite the genetic heterogeneity of ARCI, causative mutations in *TGM1* have been identified most often and reported in 55% of cases [60,62], so *TGM1* is the most preferred gene to screen for mutations in ARCI cases [20,60,63]. A latest genetic study on 19 Turkish ARCI patients was carried out by next generation sequencing that identified novel variants in ARCI associated genes of which *TGM1* was the most commonly mutated [64]. An other study reported compound heterozygous pathogenic mutations c.1187G > T and c.607C > T in *TGM1* in Chinese patients with LI [65]. Other compound heterozygous sequence variants c.327delG and c.791G > A in the *TGM1* gene have been identified in Chinese patients [52]. While applying Petrolatum, a bland emollient, regularly, a self-healing collodion Vietnamese baby was recovered, as the collodion membrane was replaced by a layer of hyperkeratosis on the trunk and limbs [66]. Although updated data is not available for the prevalence of ARCI in the Pakistani population, a doctoral study carried out on 45 ARCI patients from diverse demographic origins around the globe were invitigated, the cohort having 42% (19 patients) of patients from Pakistan [67].

Consanguineous marriages are preferred in many ethos due to socio-cultural benefits. A culture of cousin marriages has led to large family sizes which results in genetic disorders. The molecular analysis of such disorders is an attempt to verify the homozygosity as a result of interbreeding. Here, we enrolled a consanguineous family from Balochistan, Pakistan, suspected with autosomal recessive LI. Genetic analysis carryied out on genomic DNA and sequence analysis identified a nonsense sequence variant (c.131G > A; p.Trp44*) in exon 2 of the *TGM1* gene.

## 2. Materials and Methods

### 2.1. Study Subjects and Ethical Approval

**Figure 1:**
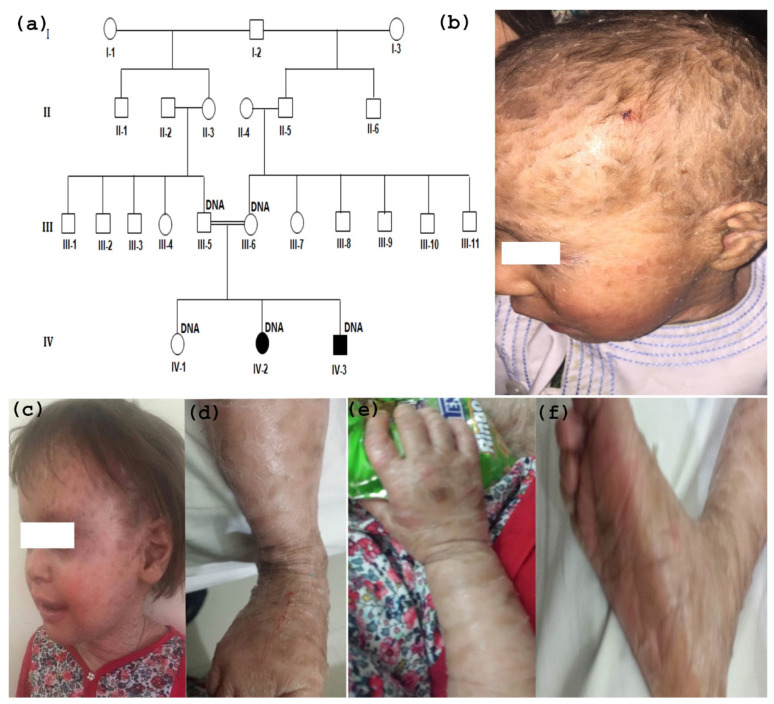
(**a**) Pedigree of a consanguineous family with lamellar ichthyosis. Affected and unaffected individuals are shown by filled and unfilled symbols, respectively. The individual numbers denoted by “DNA” indicate the sample available for this study. A double line uniting two members indicates consanguineous marriage. (**b**,**c**) Clinical features of LI patients showing thick large scales on their body including face, hyperkeratosis dry and sparse hair with scanty eyebrows, (**d**–**f**) both the patients showing keratoderma on their palms and soles and finer scales on legs and arm.

The present study was conducted according to the recommendation of the Helsinki declaration. An ethical approval was obtained from the Institutional Review Board (IRB), Balochistan University of Information Technology, Engineering and Management Sciences (BUITEMS), Quetta—Pakistan. A consanguineous family (Figure 1a) with clinical manifestations of autosomal recessive hereditary ichthyosis was ascertained from district Pishin, Balochistan. Both the affected individuals were examined by a medical expert at the local Government Sandeman Hospital Quetta, Pakistan. Written informed consent was signed by the parents of the siblings for participation in the study.

### 2.2. Clinical and Family History Interviews, Sample Collection and Extraction of Genomic DNA

Detailed clinical and medical history was taken from guardians of the affected family members to record the dermatological history of the enrolled patients. The family was also interviewed to draw a detailed family pedigree and to trace the consanguinity. Multiple family members were also interviewed to find out about skin or related disorders in the extended family. Pedigree was constructed on the information from family members. Photographs of the patients were taken after the informed written consent form. Peripheral blood samples were collected from affected and healthy family members in EDTA tubes by using disposable syringes and were stored at 4 °C during sampling. Later samples were frozen in the laboratory. In total, we collected blood samples from two affected including a female (IV-2) and a male (IV-3) and three unaffected family members (IV-1, III-5, III-6). Genomic DNA was extracted from whole blood collected from participants by using an inorganic method. In this method, 3–5 mL of blood was kept at −20 °C for about 24 h before the DNA extraction. The frozen blood was melted at room temperature and transferred to a 15 mL falcon tube. An amount of 10–12 mL of TE lysis buffer was added to the falcon tubes containing blood and mixed thoroughly. The mixture was centrifuged at 4500 rpm for 15 min and the supernatant discarded. This step was repeated 2–3 times to get a clear pellet. The pellet was suspended in 3 mL of TNE, 120 µL of 10% SDS, and 100 µL of proteinase K, and incubated overnight at 45 °C. The following day after digestion, 300 µL of 6M NaCl was added in the samples and placed in a beaker containing ice for 10 min. The falcon tubes were centrifuged for 10 min at 4500 rpm. After centrifugation, two layers were formed and the upper layer, containing the DNA, was carefully transferred into a new tube. An equal volume of isopropanol was added to the tube, kept for 10 min at room temperature, and carefully mixed several times, DNA was precipitated in isopropanol and appeared as threads in the mixture. The tubes were centrifuged at 4500 rpm for 10 min to settle down the DNA at the bottom of the falcon tubes and the supernatant discarded. The DNA pallet was washed with 70% ethanol (200 µL), and centrifuged at 4500 rpm for 5 min. The ethanol was discarded and the tube was placed overnight on tissue paper for complete evaporation of ethanol. Finally, the DNA pallet was dissolved in 300 to 500 µL of low TE buffer, depending on the size of the DNA palette. The DNA was quantified with Nanodrop-1000 spectrophotometer (Thermal Scientific, Wilmington, MA, USA).

### 2.3. Amplification and Sequencing of TGM1 Gene

All 15 exons and flanking exon-intron boundaries of the *TGM1* gene were checked for mutation screening in all the samples by Sanger sequencing and analysis. UCSC genome browser and Primer3 software were used to design forward and reverse primers for the *TGM1* gene (MIM 252300) [68]. Amplification of the entire *TGM1* gene was done along with flanking sequences of exon–intron boundaries by polymerase chain reaction (PCR). PCR reaction was performed by adding 40 ng of DNA sample to 1 µL of Taq DNA polymerase (MBI Fermentas, Sunderland, UK), 200 µM from each dNTPs, and 20 pico mol of each of forward and reverse primer mixed well with 1X PCR buffer to make a total volume of 25 µL. The sample mixture was centrifuged for 25–30 s and amplified in T3 thermocycler (Biometra, Konrad-Zuse-Straße 1, 07745 Jena, Germany). The standard PCR steps were followed as initial denaturation for 5 min at 96 °C and an amplification step of 35–40 cycles which comprised of an initial denaturation for 1 min at 96 °C, annealing at 63 °C for 1 min, and elongation for 1 min at 72 °C. The final step of extension was carried out at 72 °C for 10 min. The amplified PCR products were purified using the commercially available kit (Marligen Biosciences, Ijamsville, MD, USA). Sanger sequencing was performed by using the ABI 310 genetic analyzer, an automated DNA sequencer (Applied Biosystem, Foster City, CA, USA). Lastly, a sequence alignment tool (BioEdit) version 6.0.7 (Ibis Biosciences, Carlsbad, CA, USA) was used to search for variants in the *TGM1* gene by comparing with the reference sequence of the *TGM1* sequence (GenBank Accession Number: NM_000359). The identified variant was searched in public available databases such as dbSNP, 1000-genomes, EVS, ExAC, and gnomAD.

### 2.4. Computational Analysis

Computational tools were applied for comparative structural analysis of mutant and wild type TGase 1 proteins. The protein sequence was retrieved from UniprotKB (http://www.uniprot.org; ID P22735) (site accessed on 28 October 2021) in FASTA format. The 3D structure was modeled using first identified suitable multiple structural templates by LOMETS, a multitudinous threading approach, with atomic coordinates established through repetitive template-based splinter convoked simulations, all provided by a unified platform for protein structure prediction I-TASSER [69]. The model was energetically minimized by using the YASARA knowledge-based force field [70]. The minimized model was subjected to VERIFY3D [71], ERRAT [72], WHATCHECK [73], and PROCHECK [53] for validation. Structural comparison was done through the PyMol Molecular Graphics System v2.2.3 [54].

## 3. Results

### 3.1. Clinical Description

The pedigree details showed that the family is consanguineous, and the phenotype is segregating in the autosomal recessive mode of inheritance (Figure 1a). The family consists of one normal child (IV-1), one affected female (IV-2), and one male patient (IV-3). The age of the patients were 6 years, 3 years, and 10 months, respectively. Both the patients were diagnosed with nonsyndromic lamellar ichthyosis. Both the patients had the history of being born as collodion babies to the healthy parents. After two to three weeks, the collodion covers disappeared and additional symptoms including rough, dry, thickened, dark brown armor-like scales appeared on the whole body surface. They had clinical symptoms of hypohidrosis due to scaling that obstructs sweat gland function. The intensity of hyperkeratosis differed on different parts of body showing thicker scales on areas of thicker subcutaneous. Abnormal lips disorder (eclabium) and eversion of eyelids (ectropion) were also observed in both the affected individuals of the family. They showed dry and sparse hairs with scanty eyebrows (Figure 1b,c). Additional features including severe keratoderma in their palms and soles, and finer scales on legs and arm were present (Figure 1d–f). No other additional phenotypes, physical and intellectual development, and no history of skin disorder in their family was reported.

### 3.2. TGM1 Gene Sequencing

**Figure 2:**
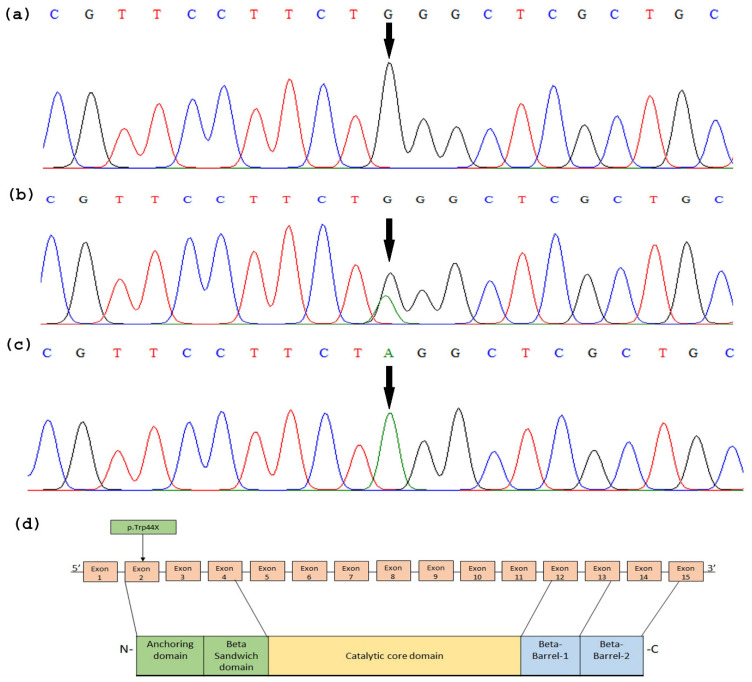
Sequence analysis of a nonsense sequence variant (c.131G > A; p.Trp44*) in exon 2 of the *TGM1* gene. The arrow indicates position of mutation. (**a**) Nucleotide sequence in the unaffected individuals, (**b**) in the heterozygous carrier, and (**c**) in the affected individuals. (**d**) Position of mutation (p.Trp44*) indicated in relationship to *TGM1* gene and Tgase-1 domains.

The *TGM1* gene was sequenced in two affected individuals and their healthy parents based on their clinical phenotypes. DNA sequencing revealed a homozygous variant Chr14: 24731428C > T leading to nonsense variant c.131G > A; p. Trp44* in exon 2 of the gene ClinVar accession number (VCV000633816.13) in two affected individuals of the family (Figure 2). It results in the substitution of tryptophan (TGG) by stop codon (UAG) at amino acid position 44 (p. Trp44*). This nonsense variant was present in a heterozygous state in their parents (III-5 and III-6). Additional sequencing was carried out in 140 control individuals outside the family where this mutation was absent. The identified mutation has not been reported in a public data basis such as the Exome Variant server, Exome Aggregation Consortium (ExAC), and genome aggregation database (gnomAD).

### 3.3. In Silico and computational Analysis

**Figure 3:**
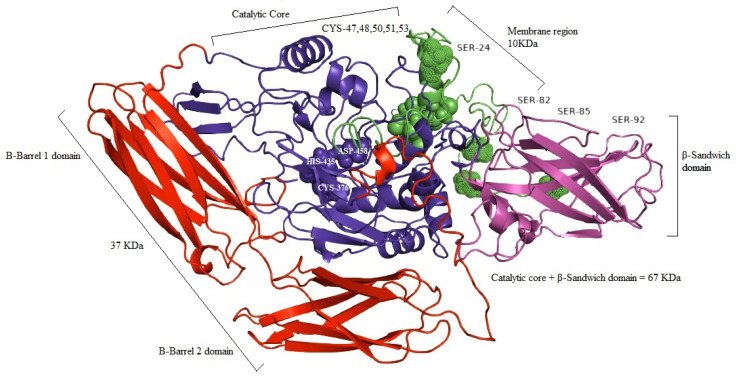
Predicted 3D model of TGase1 enzyme in Ribbon. Figure shows four main structural domains with 37Kda, B-barrel 1, and B-Barrel 2 domain in red, and 67KDa B-Sandwich domain in magenta and catalytic core domain in blue. The 10 Kda membrane anchorage part is indicated in green with SER-24, SER 82, SER-85, and SER-92; the phosphorylation sites are represented in dot, CYS-47, CYS-48, CYS-50, CYS-51, CYS-43; the proposed site of acylation is represented in spheres. The catalytic triad CYS-376, HIS-435, and ASP-458 is mentioned within catalytic core.

**Figure 4:**
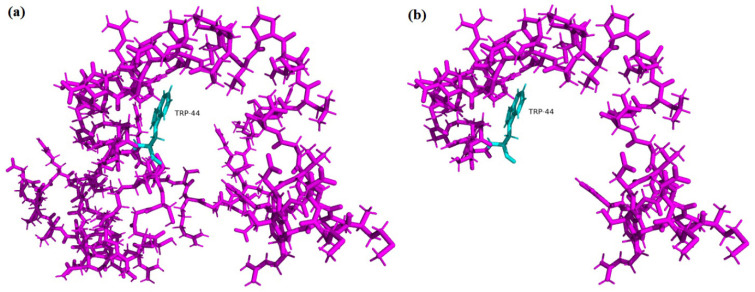
The 10 Kda membrane anchorage region (Met1-Arg92) represented in sticks. (**a**) The normal membrane anchorage region in magenta with Trp-44 in cyan; the site of nonsense mutation and (**b**) the mutant membrane anchorage region with the Trp-44 labelled.

The identified variant is checked by different *In Silico* prediction tools to see the effect of the variant on the structure of protein. Mutation Taster (http://www.mutationtaster.org/) (site accessed on 28 October 2021) predicted the variant as disease-causing. The modelled wild type TGase 1 protein structure was predicted and labeled with the domains, catalytic triad, and other important residues (Figure 3). Comparative structural analysis of the mutant protein unveiled that the 67 kDa β-sandwich domain (Ser94–Phe246) and catalytic core domain (Asn247–Arg572) that contains active site residues Cys376, His435, and Asp458 are completely lost; also the 33 kDa fragment of β-barrel 1 (Gly573–Arg688) and β-barrel 2 domain (Thr689–Ala816) are wiped out. Additionally, the 10 kDa (Met1-Arg92) domain that contains the presumed site of acylation (cysteine residues 47, 48, 50, 51, and 53) which confers membrane anchorage are lost (Figure 4). Serine residues (S24, S85, S92, and especially S82) in human TGase 1 which act as phosphorylation sites are missing with the exception of S24. The 10/67/33-kDa complex, necessary for the function of the TGase 1 enzyme, doesn’t form, resulting in a loss of almost all of its functions including the catalytic activity.

## 4. Discussion

In this study, two patients from a consanguineous family with autosomal recessive congenital ichthyosis (ARCI) were evaluated for clinical and genetic analysis. We enrolled five family members in the study including two affected individuals, a female (IV-2) and a male (IV-3), and three unaffected family members (IV-1, III-5, III-6). Both affected members showed closely related phenotypes of lamellar ichthyosis and were born as collodion babies. Their skin was covered with rough, dry, thickened, dark brown armor-like scales. Eclabium and ectropion were also observed in both the affected individuals of the family. They showed dry and sparse hairs with scanty eyebrows. The clinical presentation and medical history of the studied patients resemble the phenotypic features of a large cohort of ARCI patients from the US, Oman, Sweden, and Estonia who had collodion membrane at birth replaced by plate-like dark thickened scales along with ectropion and eclabium [55,74,75]. The collodion membrane along with ectropion is reported in 80–90% of the patients with mutations in the *TGM1* gene [50,76]. The enrolled patients presented alopecia which has been reported in other patients with *TGM1* mutations [55,74]. Another study reported that thick scales distinguish the patients with mutations in the *TGM1* gene compared to those without *TGM1*; in the latter case, the scales are fine [77]. The chances of collodion membrane, ectropion, and alopecia are four times more expected in ARCI patients with mutations in the *TGM1* gene compared to those who have mutations in other known genes causing ichthyosis [55].

Various studies revealed 242 different variants in the *TGM1* gene in patients with LI/ CIE (http://www.hgmd.cf.ac.uk/ac/ professional version) Accessed on 22 October 2022. Out of 242 variants, a majority of the variants (166) are either missense or nonsense while the remaining include splicing variants (26), regulatory site variant (1), small deletions (31), small insertions (10), small indels (6), gross deletion (1), and gross insertion (1) (Supplementary Table S1). Herein, we reported a nonsense variant (c.131G > A; p. Trp44*) in the *TGM1* gene. This is the first study reporting this variant in the population of Pakistan. This nonsense variant has been reported previously in Ichthyosis patients from Turkey and Iran who were born as collodion babies to consanguineous parents: ClinVar accession number (VCV000633816.13) [78,79]. The patient from Iran presented a characteristically severe form of the LI phenotype, having large thick plate-like scales along with palmoplantar keratoderma, ectropion, and eclabium but he did not show any sign of alopecia [79]. His clinical phenotype resembled the clinical symptoms of the present study patients except for alopecia, whereas the Turkish patient exhibited an unexpectedly less severe phenotype, diagnosed as self-improving collodion ichthyosis, having hyperkeratosis in the form of fine erythematous sclaes and mild ectropion but without eclabium and alopecia. The patient has responded to the use of topical emollients and improved the clinical phenotype [78]. Missense variants in *TGM1* have been identified in few ichthyosis patients with clinical phenotypes of self-improving collodion ichthyosis and truncated variants have caused severe forms of LI pictures [23,78]. The differences in clinical symptoms of patients with the same truncation mutation (c.131G > A; p. Trp44*) may be due to factors such as modifier genes and belonging to different regions and ethnic backgrounds.

The identified variant is located in exon 2 of *TGM1,* which support the results of the previous study reporting variants in the *TGM1* gene in ARCI patients; most of the variants were in first five exons [55,80]. This gene has a transcription start point at exon 2 which is highly conserved [27,62]. Single base substitution mutations have been reported frequently in the *TGM1* gene in which nonsense mutations are associated with a severe LI phenotype [81]. The truncating mutation p.Arg78* and p.Arg54* have been found previously in exon 2 of the *TGM1* gene [80,82]. Nonsense mutations have caused an absence of TGase-1 mRNA transcripts, enzyme levels, and catalytic activity in the epidermis of patients [60,83]. Mutation screening of *TGM1* in a consanguineous LI family of Pakistan found a novel homozygous missense mutation (c.1363T > C) [84]. Another deletion mutation (c.1084delC) in the *TGM1* gene has been found in a Pakistani family with LI patients resulting in premature truncation of the TGase-1 enzyme [85].

The TGase-1 enzyme has two domains: a beta sandwich domain and an anchoring domain in the N-terminal part. A catalytic core domain and two beta barrel domains are present in the C-Terminal part of the enzyme [27,60,86,87]. The expression of TGase-1 in the human skin produces a cornified cell envelop (CCE) which is needed for the formation of N-(c-glutamyl) lysine isopeptide bonds between different proteins, including involucrin, asloricrin, and proline-rich proteins. TGase-1 in the inner layer of skin catalyzes the binding of ω-hydroxyceramides of CCE with proteins. Three amino acids, Cys377, His436, and Asp459, aid in the catalytic activities of TGase-1 in the catalytic domain of the enzyme. The identified mutation created a premature termination codon in the N terminal part of the encoded TGase1 protein, leading to the loss of a major part of the enzyme because of its location in the catalytic core domain, and is predicted to cause premature termination of transcribed TGase-1 mRNA, resulting in a truncated polypeptide with either an absence of essential functional domains or a complete loss of enzyme due to the reduced size of mRNA by nonsense mediated mRNA decay and caused the deficient cross linking of CCE proteins.

Bioinformatics analysis revealed that conformational changes were observed in the domains, containing active sites of mutated *TGM1* (c.131G > A; p.Trp44*), which might damage the TGase 1 pathway. Abnormally functioning stratum corneum with defective intercellular lipid layers are formed as a result of this loss of enzyme activity which leads into abnormalities in skin and other clinical manifestations of LI.

Although treatment is not available for most of the genetic disorders, emollients are recommended for LI patients and should be used on daily basis. Acitretin improves keratinization, hypohidrosis, and ectropion. Use of lubricating agents, typical retinoids, and keratolytic agents are also recommended depending on overall lesions of patient [88]. Patients should have regular eye checkups, and long term use of eye lubricants to relieve ocular symptoms [89].

## 5. Conclusions

In conclusion, the present study identified a homozygous variant Chr14: 24731428C > T leading to nonsense variant c.131G > A; p. Trp44* in exon 2 of the *TGM1* gene in two affected individuals from a consanguineous family. The *TGM1* gene is most frequently mutated in patients with lamellar ichthyosis (LI); so far, 242 different genetic variants have been identified in *TGM1*. Computational analysis predicted the loss of functional domains of translated enzyme TGase-1, resulting in the complete absence of its catalytic function, causing the clinical phenotype of the studied patients. Our study has solved the molecular etiology of LI patients for the first time in our population and contributed to expand the mutation spectrum of *TGM1* in the Pakistani population. These findings will form the basis for better diagnosis, prenatal testing, and genetic counseling of LI patients in the Pakistani population.

## Supplementary Materials

The following supporting information can be downloaded at: https://www.mdpi.com/article/10.3390/medicina59010103/s1, Table S1: Reported Variants in TGM1 Gene.
